# Quantitative γ-H2AX immunofluorescence method for DNA double-strand break analysis in testis and liver after intravenous administration of ^111^InCl_3_

**DOI:** 10.1186/s13550-020-0604-8

**Published:** 2020-03-19

**Authors:** Anna Stenvall, Erik Larsson, Bo Holmqvist, Sven-Erik Strand, Bo-Anders Jönsson

**Affiliations:** 1 Department of Medical Radiation Physics, Clinical Sciences, Lund University, Lund, Sweden; 2 Department of Radiation Physics, Skåne University Hospital, Lund, Sweden; 3 ImaGene-iT AB, Lund, Sweden

**Keywords:** DNA damage, γ-H2AX, Absorbed dose, Internal irradiation, ^111^In, Tissue level, Testis, Liver

## Abstract

**Background:**

It is well known that a severe cell injury after exposure to ionizing radiation is the induction of DNA double-strand breaks (DSBs). After exposure, an early response to DSBs is the phosphorylation of the histone H2AX molecule regions adjacent to the DSBs, referred to as γ-H2AX foci. The γ-H2AX assay after external exposure is a good tool for investigating the link between the absorbed dose and biological effect. However, less is known about DNA DSBs and γ-H2AX foci within the tissue microarchitecture after internal irradiation from radiopharmaceuticals. Therefore, in this study, we aimed to develop and validate a quantitative ex vivo model using γ-H2AX immunofluorescence staining and confocal laser scanning microscopy (CLSM) to investigate its applicability in nuclear medicine dosimetry research. Liver and testis were selected as the organs to study after intravenous administration of ^111^InCl_3_.

**Results:**

In this study, we developed and validated a method that combines ex vivo γ-H2AX foci labeling of tissue sections with in vivo systemically irradiated mouse testis and liver tissues. The method includes CLSM imaging for intracellular cell-specific γ-H2AX foci detection and quantification and absorbed dose calculations. After exposure to ionizing radiation from ^111^InCl_3_, both hepatocytes and non-hepatocytes within the liver showed an absorbed dose-dependent elevation of γ-H2AX foci, whereas no such correlation was seen for the testis tissue.

**Conclusion:**

It is possible to detect and quantify the radiation-induced γ-H2AX foci within the tissues of organs at risk after internal irradiation. We conclude that our method developed is an appropriate tool to study dose–response relationships in animal organs and human tissue biopsies after internal exposure to radiation.

## Introduction

It is well established that when mammalian cells are exposed to ionizing radiation, the most severe cell injury is the induction of DNA double-strand breaks (DSBs), which often results in cell death but can also initiate genomic instability [1]. An early response to DSBs is the phosphorylation of the histone H2AX at serine 139, referred to as γ-H2AX foci. These discrete foci appear specifically at sites of the induced DNA double-strand breaks and represent the repair processes of DSB lesions [1, 2]. Using antibodies against phosphorylated H2AX, foci can be detected and visualized by immunofluorescence labeling for analyses with fluorescence microscopy and confocal laser scanning microscopy techniques (CLSM) [3, 4].

The absorbed dose necessary for observation of irradiation-induced foci depends on the endogenous level of breakage/foci. Several pioneering studies of irradiation-induced γ-H2AX foci were performed using acute external irradiation on fibroblasts and lymphocytes, where the endogenous levels of foci are low [5]. Furthermore, the use of γ-H2AX detection for quantification of irradiation effects from acute single or split exposure by external beam irradiation within various cells in vitro [6, 7] and in tissues after in vivo irradiation [8–11] has been reported. However, studies on the presence of γ-H2AX foci after continuous internal irradiation after systemic administration of radiopharmaceuticals used for therapy have been confined to leukocytes in the peripheral blood cells [12, 13] and mice kidneys [14]. Hence, it is currently unclear how DNA DSBs are distributed within differentiated mammalian tissue after internal irradiation originating from radionuclides or radiopharmaceuticals and how different tissues within normal organs respond.

In this study, two different tissues were selected: testicular tissue, because of its high proliferating rate and high radiosensitivity; and liver tissue, because of its normally non-proliferating rate and low radiosensitivity [15]. Indium-111 chloride, ^111^InCl_3_, was selected as the radioactive compound of interest based on earlier basic investigations that demonstrated uptake of ^111^In into the testes and into developing germ cells in both rodents [16–18] and humans [19, 20]. In addition, ^111^In as a radiometal is trapped by transferrin in the blood plasma, forming metal protein complexes. These are transported to several tissues, including the testes and the liver, with especially noticeable accumulation in the liver and bone marrow [21], resulting in high ^111^In uptake in the liver [17, 22].

### Testis

Mammalian spermatogenesis is a steady-state system with high proliferation activity, in which the differentiated spermatogonia are the most sensitive cells in adult humans and are responsible for the radiation-induced reduction of DNA-synthesizing testicular cells [23, 24]. Thus, the testis is considered a critical tissue in both diagnosis and therapy with ionizing radiation. Temporary sterility is observed after an absorbed dose of external radiation as low as ≈ 0.15 Gy from external irradiation [15, 25, 26]. The accumulation of radionuclides and radiopharmaceuticals in the testis may also lead to permanent sterility owing to depletion of the proliferating tissue [19, 27]. The radiobiological response of testicular germ cells post exposure to ionizing radiation has been investigated in mice and rats [28–31] and is considered relevant to humans [24]. Consequently, spermatogenesis has been used as an in vivo model for radiobiological research [18, 32, 33], and it has been suggested as a biological dosimeter of ionizing radiation [34].

H2AX phosphorylation and the formation of γ-H2AX foci in the testis are not restricted to radiation effects. The formation of haploid germ cells requires the formation and repair of meiosis-specific DNA DSBs through programmed changes in the chromatin structure [35, 36]. The phosphorylation and dephosphorylation of H2AX throughout the different stages of the spermatogenic cycle creates specific staining patterns correlated to germ cell development [36]. In a detailed study of the mouse testis, Hamer and coworkers [3] described the presence of γ-H2AX immunoreactivity independent of irradiation, which was present in all intermediate and B spermatogonia and in different stages of spermatocytes. However, the study also proposed that radiation-induced γ-H2AX foci were generated in specific cell types, most notably in spermatogonia, spermatocytes, and round spermatids.

### Liver

From a clinical perspective, the liver is considered a rather radio-resistant organ. A 5% incidence of radiation-induced liver disease (RILD) has been observed after fractionated external beam radiotherapy up to a mean absorbed dose to the whole liver of 30–35 Gy [15, 37, 38]. For external irradiation of one third of the liver volume, the absorbed dose for a 5% incidence of RILD is increased to > 42 Gy [39]. The cellular turnover of liver cells, the hepatocytes, is normally slow, and they do not divide under normal conditions, hence clonogenic cell death does not reflect the observed acute radiation injuries of the liver. The pathological features of RILD is a condition called veno-occlusive disease, which manifests as centrilobular congestion and necrosis [15].

The formation of γ-H2AX foci within the liver of mice was previously reported after whole-body X-ray irradiation up to absorbed doses of 5 Gy. A pronounced increase in the percentage of γ-H2AX-positive cells was identified 1 h after irradiation, increasing from 5% pre-irradiation to 26% post irradiation. The percentage of γ-H2AX-positive cells within the liver reverted to its control level 24 h after irradiation [11].

### Dosimetry

Concerning absorbed dose–effect relationships of radiopharmaceuticals, it is conventionally assumed that a radiopharmaceutical is homogeneously distributed within the source region, giving a uniform deposition of the radiation energy within the target region. However, it is known that the absorbed dose to individual cells depends on the microscopic distribution of the radioactivity [40–43]. Thus, a recurring important question in internal radiation dosimetry research is the link between the quantity of the absorbed dose and certain biological effects in the volume of energy deposition. Recently, Larsson et al. developed a small-scale anatomical model for detailed testis dosimetry [41] that permits the calculation of the absorbed dose to spermatogonia in the testicular tissue from radionuclides used in nuclear medicine, with an optional choice of source regions in the interstitial tissue or in the germ cells in the seminiferous tubules. A corresponding model for detailed liver dosimetry was developed by Stenvall et al. [42], which enabled absorbed doses to be calculated for the different microstructures within the liver tissue from optional source regions. These small-scale dosimetry models, in combination with a suitable radiobiological endpoint, may be used for a better understanding of biological lesions in the cells caused by a certain absorbed dose.

This investigation aims to develop and validate an ex vivo method for identifying and quantifying DNA double-strand breaks induced within the testis and liver after systemic administration of ^111^InCl_3_. For the analysis, we developed protocols for γ-H2AX-imunifluorescence labeling of sections from mouse tissues, microscopy imaging, and image data analysis with confocal laser scanning microscopy, as well as protocols for cell-specific quantitative digital image analyses of labeled cell nuclei and intranuclear foci.

## Materials and methods

### Study design

Twelve young adult nude mice NMRl-NU (Charles River, Germany) weighing 30–40 g were used in this study. The procedures followed in this study were approved by the local Animal Ethical Committee (approval M255-10). The animals were housed under conventional, controlled standard conditions. They were acclimatized to laboratory conditions for 1 week prior to the beginning of the experiment. They were divided into two main groups: 8 mice were injected with ^111^InCl_3_ (irradiated) and 4 were not (exposure controls). The exposed groups were intravenously injected with 63 ± 2.3 MBq (average ± standard deviation (SD)) of ^111^InCl_3_ (Mallinckrodt Medical, Petten, Holland). Four animals were sacrificed 4 h (3.7 h ± 0.1 h) post-injection (P.I.) and four animals 25 h (25.2 h ± 1 h) P.I. by cervical dislocation. The testes and the liver were then resected. One testis per animal and one lobe of the liver were transferred to pre-weighed plastic tubes and measured for activity in a well-type NaI(Tl) gamma counter (Wallac Wizard 1480, Perkin Elmer). The other testis and one additional lobe of the liver were divided into two parts and immersed in freshly made paraformaldehyde (4%) for 20 h at 4 °C.

### Dosimetry

The dosimetry calculations were based on the biokinetic data obtained from the activity content in the testis and liver of the sacrificed animals. Volume correction calibration factors were applied to the gamma counter output to compensate for differences in the measured organ volume. The measured activity (Bq) was time corrected to the time of sacrifice. The percent injected activity per gram tissue (%IA/g) was calculated for the different organs, and a mono-exponential elimination was assumed between the two time points. The effective decay constant of ^111^InCl_3_ for the organs was calculated, from which the residence times for the two exposure times were determined. The activity in the organs at the time of injection (*A*_0_) was calculated and used in combination with the respective residence time (*τ*_*h*_) and the precalculated S factors for the self-dose component [44] based on the anatomical small-animal phantom, MOBY [45], to obtain the mean absorbed doses at the two different time points (${\overline{D}}_h$) according to Eq. 1.

1 $$ {\overline{D}}_h={A}_0\bullet {\tau}_h\bullet {S}_{\mathrm{self}\ \mathrm{dose}} $$

### γ-H2AX immunofluorescence labeling

The specimens were rinsed three times for 5 s each in phosphate buffer saline (PBS 0.1 M, pH 7.4) and dehydrated in a graded alcohol (EtOH) series (70–100%). They were then further immersed in equal volumes of ethanol (100%) and xylene (100%), followed by xylene (100%). The testes and the liver lobes were then infiltrated with 100% paraffin for 1 h at 58 °C, followed by immersion in fresh (100%) paraffin at 58 °C overnight. From the paraffin blocks, consecutive sections were made on a rotation microtome (Rotary Microtome, HM 360, Microm International GmbH, Waldorf, Germany). Five micrometer-thick sections of the testis and liver tissues were collected on SuperFrost Plus microscope slides (G Menzel, Braunschweig, Germany) and dried for 16–18 h at 37 °C. The sections were then deparaffinized, starting with immersion in 100% xylene, followed by immersion in a graded alcohol series down to 70% EtOH. Antigen retrieval was performed by immersing the slides in citrate buffer (pH 6.0) containing a detergent (0.5% Tween 20) that was heated to 90 °C for 10 min. The slides were allowed to cool to room temperature (RT, around 20 °C), incubated in acetone (100%, about 5 s), and then rinsed in PBS three times for 5 min each.

For γ-H2AX immunofluorescence labeling, two different primary antibodies against γ-H2AX were used: one mouse monoclonal anti-phospho-histone H2A.X, (clone JBW301, Merck Millipore, Darmstadt, Germany) and one rabbit polyclonal anti-γ-H2AX (Thermo Scientific Art no. PA1-25001). First, the sections were encircled with a silicon pen and immersed three times for 5 min each in a washing solution of PBSTX (0.1%) (phosphate buffer saline 0.1 M, pH 7.5, 0.1% Triton X-100) at RT. The slides were incubated with a blocking solution of PBSTX (0.1%) containing 1% bovine serum albumin (BSA) for 60 min at RT. The primary anti-γ-H2AX antibodies were diluted in 1% BSA/PBSTX (0.05%) to a concentration of 1 μg/mL for the monoclonal and 1.25 μg/ml for the polyclonal. Sections were incubated with the γ-H2AX antibody solution in a moisture chamber for 16–18 h at 4 °C. Randomly selected sections from non-exposed and exposed animals were incubated without the primary antibodies, i.e., they were used as specificity controls of the γ-H2AX labeling and secondary antibody binding. Sections were then rinsed in PBS three times for 5 min each under gentle shaking.

To visualize the primary antibody binding sites, sections were incubated with secondary goat antibodies against mouse IgG conjugated with Alexa Fluor 488 (Jackson Immunoresearch, Baltimore, MD, USA) or against rabbit IgG conjugated with Alexa Fluor 568 (Invitrogen. Art no. A11036). Secondary antibodies were diluted 1:150 (13.3 μg/mL) in 1% BSA/PBS, and sections were incubated for 60 min at RT. Sections were then rinsed in PBS three times for 5 min each and were incubated with 4′,6-diamidino-2-phenylindole (DAPI, nuclear labeling, Invitrogen, USA) at a concentration of 0.1 μM for 20 min at RT. Sections were then rinsed with PBS for 5 min before being mounted and coverslipped using DAKO fluorescent mounting medium (Carpenteria, USA).

### Laser confocal scanning microscopy and image acquisition

The γ-H2AX foci labeling was first analyzed with a wide-field epi-fluorescence microscope (Olympus AX60) and then with a confocal laser scanning microscope (CLSM, Zeiss 510 Meta, Dept. Biology, Lund University). Both these were equipped for the excitation and emission wavelengths of Alexa Fluor 488, Alexa Fluor 568, and DAPI.

Digital images for qualitative and quantitative image analyses were sampled. For the testis, two tissue sections from each animal were scanned with a × 20/0.8 Plan Apochromat objective for an overview of the labeling. From each section four tubuli profiles were selected (x/y stage position marked and stored by the acquisition software ZEN 2009), where three-dimensional (3D) images (z-stacks) were obtained with the × 63/1.4 objective. Cells were histologically and morphologically defined according to their location and DAPI nuclear labeling, and the scanning position was chosen as to include spermatogonia (type A and B) and primary spermatocytes. For the liver, two tissue sections from each animal were scanned. First, the area was scanned with a × 20/0.8 Plan Apochromat objective to achieve an overview of the labeling, whereupon one identifiable liver lobule was randomly chosen for the acquisition of two z-stacks, one close to the central vein of the liver lobule and one close to the portal tract. Cells were defined as hepatocytes or non-hepatocytes (including sinusoidal endothelial cells, Kupffer cells, hepatic stellate cells).

Confocal settings were optimized individually for the testis and liver tissues to maximize the detection of foci. For the testis, the tubule with the highest fluorescence intensity at × 20/0.8 was selected as reference, and the acquisition settings (laser power, photomultiplier tube (PMT) detector gain, digital offset) for the × 63/1.4 objective z-stacks were optimized for this profile. For the liver, a reference tissue section from an animal exposed for 4 h was used. The acquisition settings from the testis samples were initially applied. To optimize the detection of foci, the laser power and PMT detector gain were increased for the γ-H2AX-positive channel. Z-stacks for both testis and liver were acquired with a 1024 × 1024 matrix size (x/y pixel width 140 nm, z-step size 581 nm, i.e., 10 optical sections per stack) using a pixel dwell of 3.20 μs per pixel and channel.

### Quantification of γ-H2AX foci from CLSM z-stacks

The digital image data of nuclei were used to calculate the number of γ-H2AX foci per nuclei and compare the exposed and non-exposed animals. The CLSM image analysis was performed in a cell-specific manner. For the spermatogonia and primary spermatocytes, data were collected from a minimum of 30 (between 30–110) cells per animal from transversal sections from different regions of the tubules. For the hepatocytes and non-hepatocytes, data were collected from two regions with a minimum of 30 (between 30–70) cells per region.

#### Testis

For the image analysis of the testis tissue z-stacks, the Image J software (NIH, USA) [46] was used to analyze the number of γ-H2AX foci in spermatogonia (type A and B) and primary spermatocytes nuclei. Quantification was performed in the γ-H2AX-positive channel only and the regions of interest (ROIs) containing the nuclei of interest were manually identified. In the 3D z-stack images, the foci were segmented using the built-in function 3D Foci Picker [47]. The γ-H2AX foci were defined as coherent regions larger than 20 voxels with only one local maxima center defined by one pixel or a group of continuous pixels having the same intensity. The background intensity around each identified focus was defined by the voxels at a radius of six voxels from each local maximum. We obtain the average value of foci per nuclei for each z-stack by dividing the total number of γ-H2AX foci within the z-stack volume by the number of cell nuclei.

#### Liver

The liver tissue z-stacks were used to analyze the number of γ-H2AX foci within the hepatocyte and non-hepatocyte nuclei, including all nuclei present within the image. The analysis was performed using the Image J software (NIH, USA) [46]. Quantification was performed on the 3D z-stack images using both the DAPI- and γ-H2AX-positive channels. But first, a two-dimensional (2D) mask to delineate the outer border of each nuclei was created, from 2D maximum intensity projection (MIP) of the DAPI channel. The mask was based on an automatically set threshold; however, the mask was user adjustable in order to segment all pixels belonging to nuclei and exclude the background pixels. Then, the mask was filtered by a median filter with a 2-pixel radius, replacing the center pixel with the median value, whereupon watershed segmentation [48] was applied to separate adjacent identified regions. Potential empty regions within the nuclei outline were filled using the “Fill holes” algorithm, which identifies and replaces regions with background values within the nuclei outline. These outlines were transferred to the 3D z-stack of the γ-H2AX-positive channel, defining the regions wherein the γ-H2AX foci were segmented using the built-in function 3D Foci Picker [47]. The γ-H2AX foci were defined as coherent regions larger than 8 voxels with only one local maxima center defined by one pixel or a group of continuous pixels having the same intensity. The background intensity around each identified focus was defined by the voxels at a radius of six voxels from each local maximum. In the analysis, the two cell types, hepatocytes and non-hepatocytes, were manually defined according to their shape and DAPI labeling and sorted into the two groups. Data is presented both as the number of foci per nucleus and as the average values of the number of foci per nuclei for each treatment group. The z-stacks collected close to the central vein and close to the portal tract were considered equally good representatives for the normal liver parenchyma and were therefore not separated during the analysis.

### Statistical analysis

Data was tested for statistically significant differences between groups using one-way ANOVA, followed by Tukey HSD post hoc test. Statistical analysis was performed using the R software [49].

## Results

### Absorbed dose

The biokinetic data used for the absorbed dose calculations are presented in Table 1.

**Table 1:** Overview of the biokinetic data used as input for dosimetry calculations

	%IA/g at the time of sacrifice		Effective half-life (h)	Dose rate (mGy/h)		Absorbed dose per injected activity (mGy/MBq)		
	4 h P.I.	25 h P.I.		4 h P.I.	25 h P.I.	4 h P.I.	25 h P.I.	Infinity
Testis	0.36	0.28	58.7	4.6	3.8	0.31	1.65	6.5
Liver	7.63	7.01	67.9	117	102	7.75	49.6	207.1

Assuming homogenous activity distribution in the source organ, the average self-absorbed doses for animals exposed for 4 h and 25 h were 20 mGy and 0.1 Gy, respectively, to the testis and 0.5 Gy and 3.20 Gy, respectively, to the liver.

### Immunofluorescence labeling–qualitative analyses

#### Testis

**Fig. 1:**
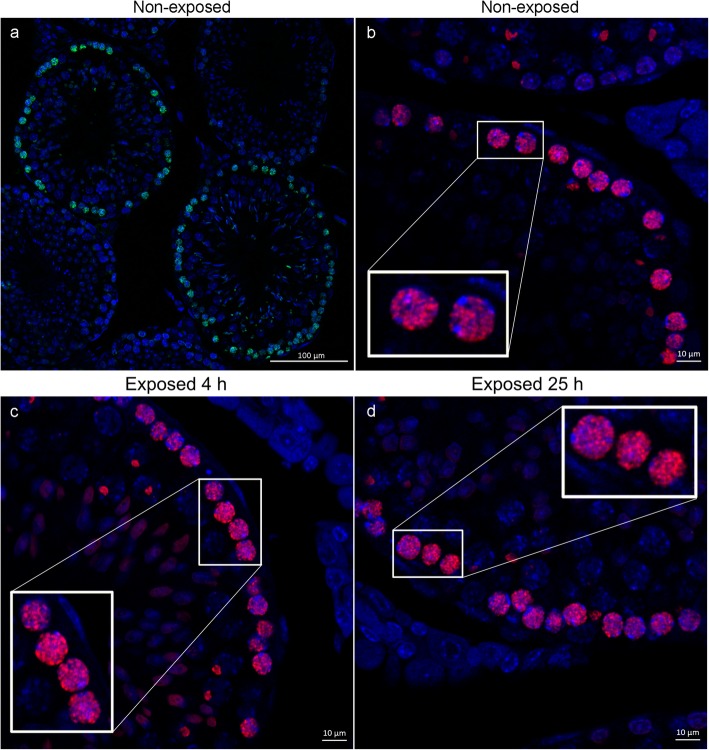
Confocal laser microscopy of testis sections. Representative images of one optical section of **a**, **b** non-exposed animals, **c** animal 4 h P.I., and **d** animal 25 h P.I. The low-magnification image in **a** illustrates the distribution of γ-H2AX labeling (green, visualized with anti-mouse AF488 conjugated secondary antibodies) and images **b**–**d** illustrate in higher magnification the γ-H2AX labeling (red, visualized with the anti-rabbit AF568 conjugated secondary antibodies). Images were adjusted for brightness and contrast. Scale bars in **a** 100 μm and in **b**–**d** 10 μm

Confocal laser scanning microscopy images of the testis provided a high signal-to-noise ratio of the intranuclear γ-H2AX foci labeling. Visual analysis of CLSM images of the testis did not reveal any discernable differences in the amount of γ-H2AX foci when comparing non-exposed and exposed animals. Many γ-H2AX-positive cells were spermatogonia (type A and B) and primary spermatocytes, defined from their size, nuclear appearance, and location next to the inner tubular wall of the seminiferous tubules (Figs. 1a–d). Thus, the distribution of different γ-H2AX-positive cell stages correlated well with previously published results [3]. Sections used for specificity control were all negative. The two different primary antibodies showed indistinguishable labeling results, supporting the specific labeling of γ-H2AX.

#### Liver

**Fig. 2:**
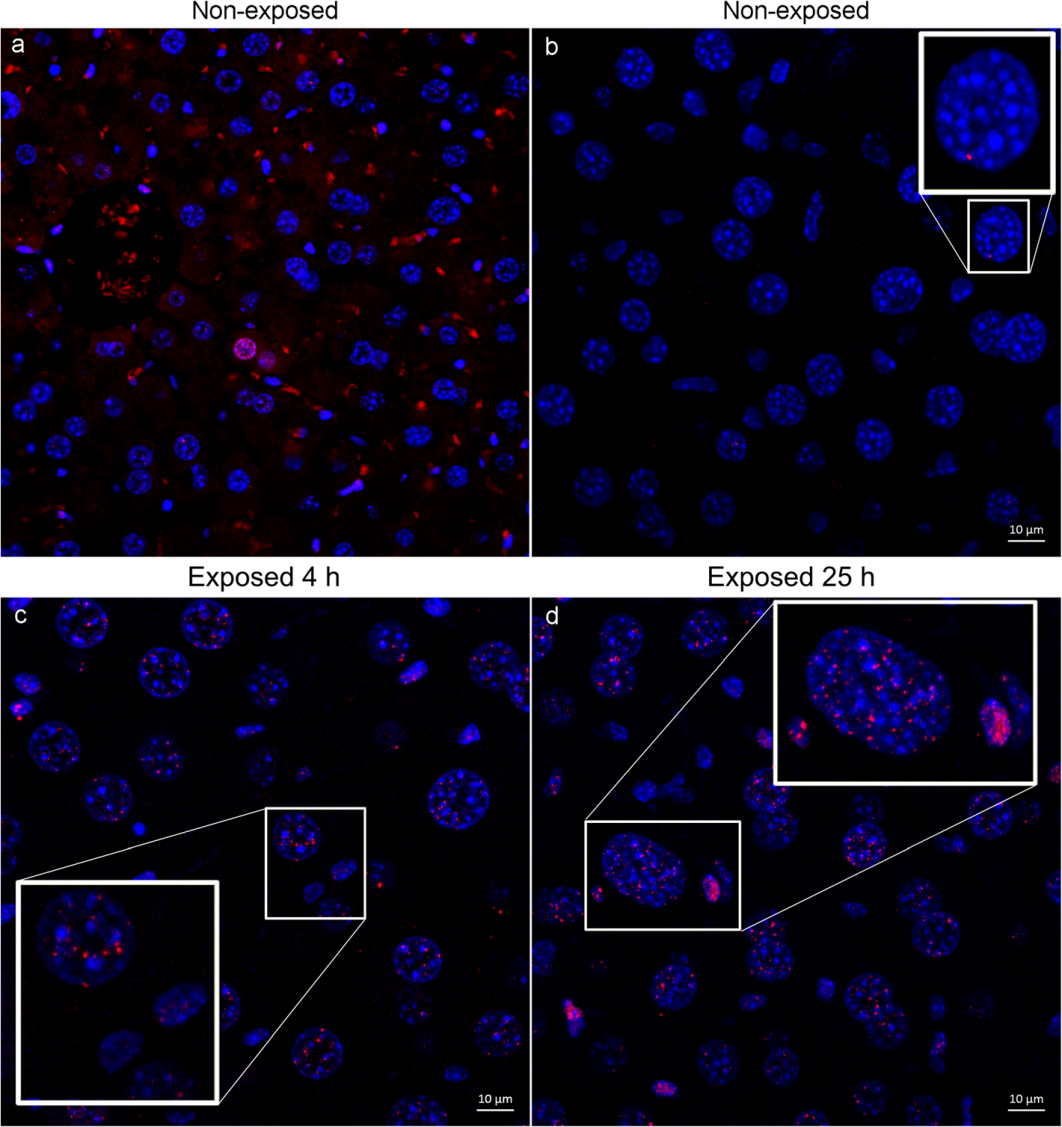
Confocal laser microscopy of liver sections. Representative illustration of images from **a**, **b** non-exposed animals, **c** animal 4 h P.I., and **d** animal 25 h P.I. Overview image in **a** taken with wide-field fluorescence microscope and in **b**–**d** with CLSM. Note the difference between the hepatocyte nuclei, which morphologically are large, with a circular cell nuclei and bright areas of heterochromatin regions compared with the non-hepatocyte nuclei, which are more elongated in shape and have more homogeneous nuclear staining. Images were adjusted for brightness and contrast. Scale bars in **b**–**d** 10 μm

High-magnification confocal laser scanning microscopy images of the liver provided a high signal-to-noise ratio of the relatively small and distinct intranuclear γ-H2AX-labeled foci compared with the testis. Visual inspection revealed a pronounced difference between the non-exposed and exposed animals. The non-exposed animals exhibited a few scattered γ-H2AX-positive nuclei, with only a single labeled focus per scanned field of view. In the livers of exposed animals, both the number of labeled nuclei and the number of intranuclear foci per cell increased by a significant order of magnitude compared with non-exposed animals, in terms of both hepatocytes and non-hepatocytes (see Fig. 2a).

### Quantitative CLSM image analyses of intranuclear γ-H2AX foci

**Fig. 3:**
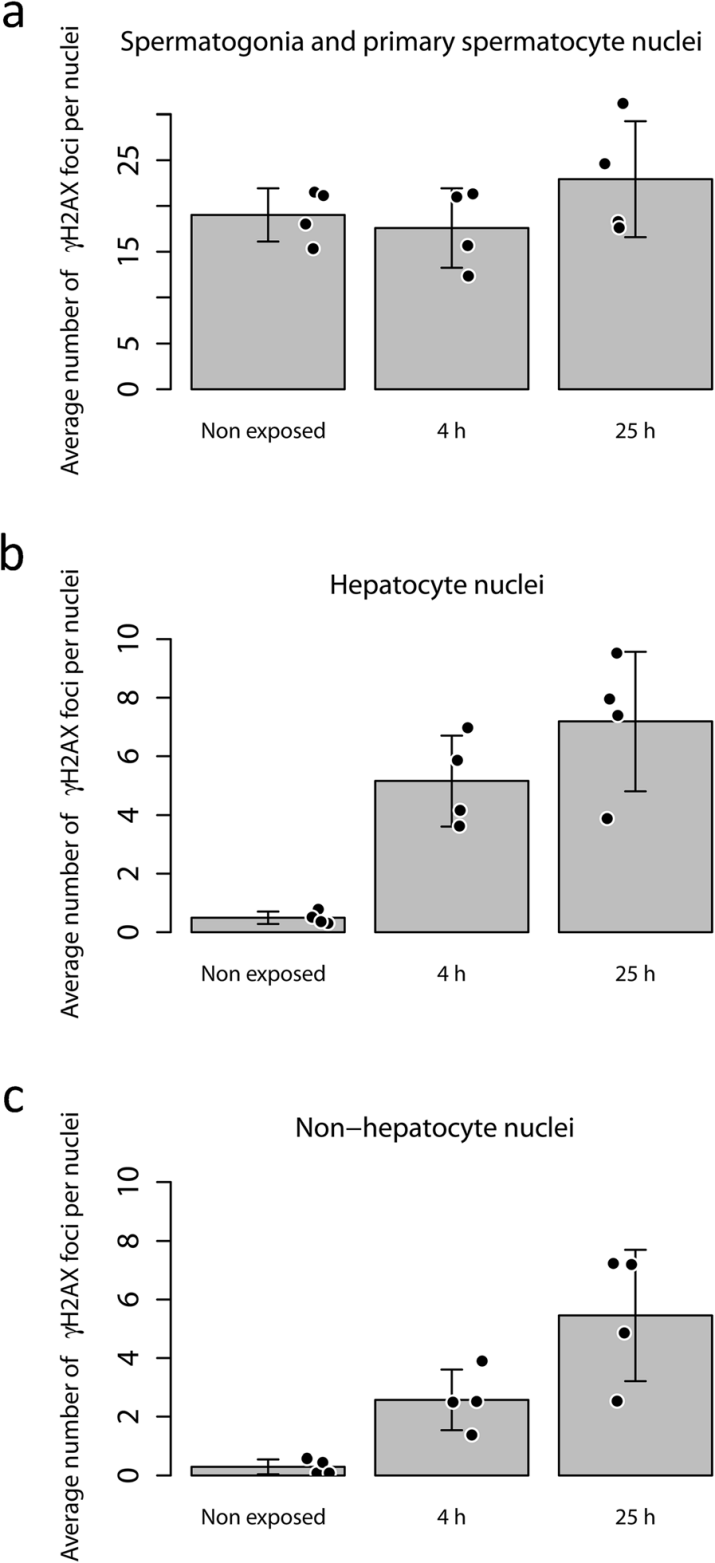
Bar plots illustrating the average number of γ-H2AX foci per cell nuclei for different groups of animals (non-exposed, 4 h P.I., and 25 h P.I.) for the **a** spermatogonia and primary spermatocytes, **b** hepatocytes, and **c** non-hepatocytes. The mean value for each individual animal within a group is illustrated by a black dot. The points have been randomly displaced along the horizontal axis to increase visibility; it should not be interpreted as a variation in time after injection

The analysis of the number of γ-H2AX foci per cell nuclei, for the testis, exhibited no statistically significant difference between any of the groups. The average number of foci per nuclei for the non-exposed control animals was 19 ± 3 (average ± SD). For the exposed animals, the average number of foci per nuclei was 18 ± 4 and 23 ± 6 for animals exposed for 4 h and 25 h, respectively (see Fig. 3a).

For the liver, the quantitative analysis of the number of γ-H2AX foci within the hepatocyte nuclei exhibited a statistically significant difference between non-exposed animals and exposed animals (regardless of exposure time) (one-way ANOVA, *F* = 17.5, *p* < 0.0008; post hoc Tukey HSD, *p* < 0.007 (4 h) and *p* < 0.0007 (25 h)). The highest average number of foci per nuclei was observed for animals exposed for 25 h (7 ± 2) and the lowest number of foci per nuclei for the control animals (0.5 ± 0.2). A significant difference was not observed between the group exposed for 4 h (average number of foci per nuclei 5 ± 2) and 25 h (post hoc Tukey HSD, *p* < 0.24) (see Fig. 3b).

For the non-hepatocytes, the group exposed for 25 h had significantly more foci per nuclei than the control animals and the group exposed for 4 h (one-way ANOVA, *F* = 13, *p* < 0.002; post hoc Tukey HSD, *p* < 0.002 (control) and *p* < 0.05 (4 h)) had the highest average number of foci per nuclei for animals exposed for 25 h (5 ± 2) and the lowest average number of foci per nuclei for the control animals (0.3 ± 0.3). There was no significant difference in the average number of foci per non-hepatocyte nuclei between non-exposed animals and animals exposed for 4 h (3 ± 1) (see Fig. 3c).

**Fig. 4:**
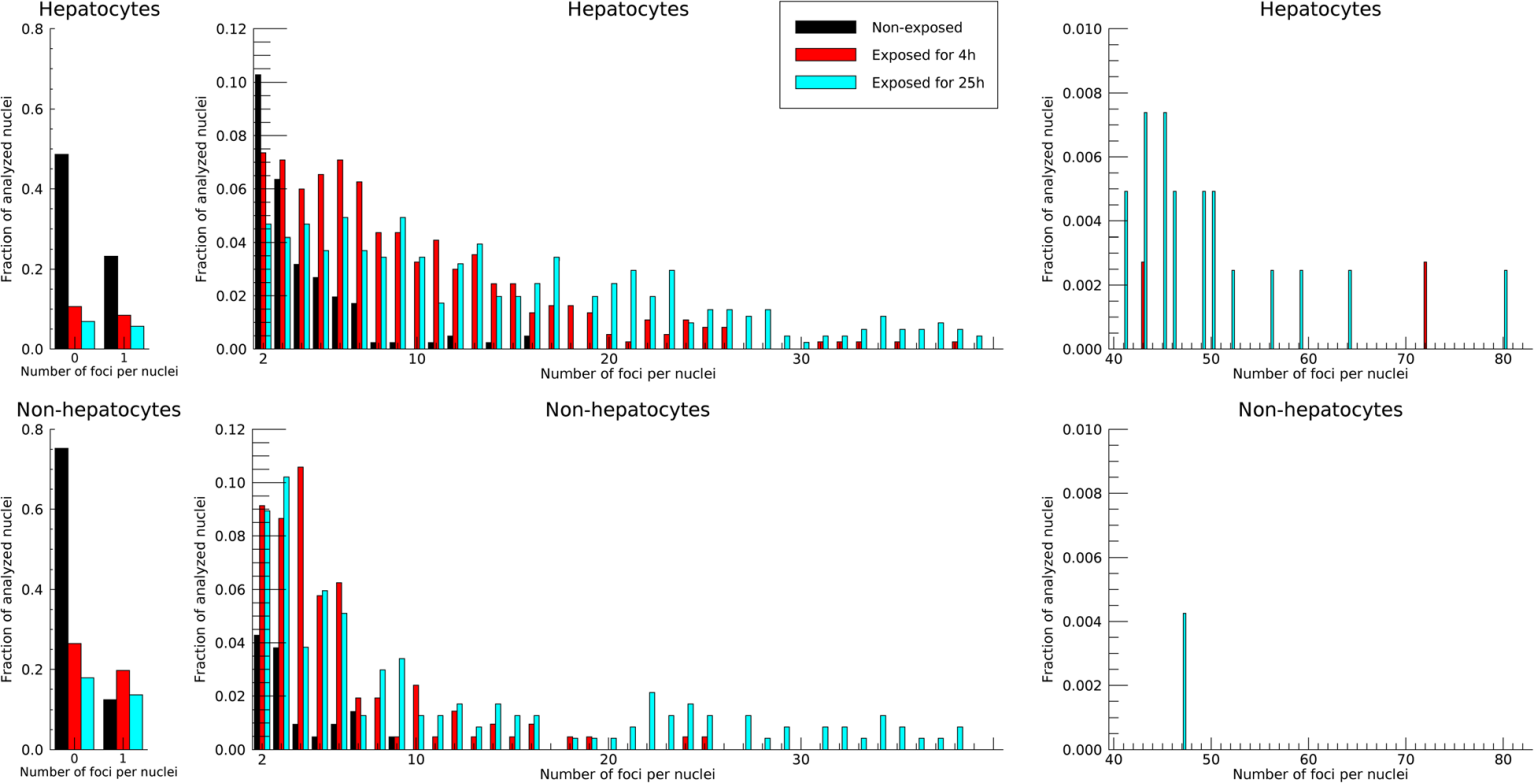
Histograms showing the fraction of analyzed nuclei containing a certain number of γ-H2AX foci for the three different groups of animals: non-exposed (black), exposed for 4 h (red), and exposed for 25 h (cyan). Note that the *x*-axis is continuous over the three horizontal sub-pictures, but splitted up with respect to the varying scale of the *y*-axis

To further evaluate the differences in average number of γ-H2AX foci per cell nuclei for the exposed and non-exposed liver cells, the fraction of analyzed nuclei containing a certain number of γ-H2AX foci was summarized through histograms (Fig. 4). With increasing exposure times, the distribution of the number of foci per nuclei increased for both hepatocytes and non-hepatocytes. However, some nuclei in the exposed groups contained only a few or no foci at all. For non-exposed animals, 49% of the analyzed hepatocytes did not contain any foci at all, whereas after 4 h of exposure, 11% of the hepatocytes contained no foci. After 25 h exposure, only 7% of the hepatocytes contained no foci. For the non-exposed non-hepatocytes, 75% of the nuclei did not contain any foci. After exposure, the percentage of non-hepatocytes that contained no foci decreased to 26% and 18% after exposure for 4 h and 25 h, respectively.

## Discussion

The potential applicability of γ-H2AX foci labeling, detection, and quantification in cells and tissues after acute external irradiation has previously been demonstrated both in vitro and ex vivo, as presented in the introduction. However, the distribution of DNA double-strand breaks within the tissue microarchitecture of liver and testis after internal irradiation from a radionuclide or radiopharmaceutical has not been previously shown. Our study proposes a suitable ex vivo method that enables γ-H2AX foci immunolabeling of internally irradiated tissue sections combined with confocal laser scanning microscopy imaging that provides quantitative digital analysis of intranuclear γ-H2AX foci. In the current analysis of tissue sections, we demonstrate an absorbed dose-dependent elevation of γ-H2AX foci after internal exposure from ^111^InCl_3_. The method was validated by using two tissues with major differences in proliferation rate and radiosensitivity, the testis and the liver. The spermatogenesis has for long and repeatedly been used as a suitable in vivo experimental model in basic radiobiological research [18, 31–34]. However, one disadvantage is that most models are based on the long-time cycle of spermatogonial cells becoming whole spermheads, to find the minimum spermhead count in survival studies as a measure of the depletion of the proliferating tissue of the testis. The novel ex vivo γ-H2AX method developed and evaluated in the present study could further expand the testis as a suitable experimental model. Moreover, our results in the liver do indicate that the ex vivo method may be suitable for analyses of induced γ-H2AX foci in other tissues.

As expected, the endogenous level (background) and variation of intranuclear foci for non-exposed animals was significantly lower in the liver cells than in the spermatogonia and primary spermatocytes. For hepatocytes and non-hepatocytes, the average number of endogenous foci per nuclei was only 0.5 ± 0.2 (mean ± SD) and 0.3 ± 0.3, respectively, whereas for the spermatogonia and primary spermatocytes, the average endogenous value of foci per nuclei was high, 19 ± 3, in accordance with published studies [3, 35, 36].

In hepatocytes, we illustrated a significant increase in the number of γ-H2AX foci per nuclei occurred after exposure. In contrast, there were no differences in the γ-H2AX foci count between exposed and non-exposed animals in the nuclei of spermatogonia or primary spermatocytes in the testis. This is consistent with the fact that a lower endogenous level of γ-H2AX foci and a higher absorbed dose, as seen in the liver, leads to increased separation between exposed and non-exposed animals [5]. The results also concur, with the conclusion by Firsanov et al. [11], that the efficiency of γ-H2AX foci formation correlates with the proliferation capabilities of the tissues.

It is known that the dose rate of the irradiation and the time point after an exposure will affect the measured number of γ-H2AX foci. Continuous radiation emitted from radionuclides, where the dose rate usually is low compared with acute exposures, will provide time for repair of sub-lethal DNA-damage to occur during the time of irradiation. This will cause a lower foci-count directly after a protracted exposure than after a single high-dose rate exposure with the same total absorbed dose. However, the foci count after a 24 h protracted irradiation is expected to be higher than 24 h after an acute exposure, as shown by van Oorschot et al. [50]. In addition, the magnitude of the dose-rate effect does vary between cell types, due to the inherent radiosensitivity, in our study represented by two contrasting tissues, related to the degree of differentiation and proliferation but also the repair time of sub-lethal DNA damage. Cells with a high inherent radiosensitivity and a slow repair rate of sublethal damage will be less affected by the dose rate effect [51]. Kühne et al. [6] measured the maximum number of γ-H2AX foci in human fibroblasts 3 min after 2 Gy X-ray irradiation. However, the foci at that time were small, and the authors suggested performing quantification 15–30 min post-exposure, despite the risk that some foci may be repaired during that time. Within the liver, Firsanov et al. [11] showed a decrease in the fraction of γ-H2AX-positive nuclei from 1 to 24 h after acute X-ray irradiation, indicating inception of DNA DSB repair after X-ray irradiation. When DNA repair mechanisms are activated, a smaller induction of γ-H2AX foci per unit absorbed dose was shown in a split dose experiment by Mariotti et al. [7], where the time between the two fractions was less than 12 h. Hence, it follows that the correlation between absorbed dose and the number of γ-H2AX foci after and between exposures is complex and will depend on the tissue of interest, the time point studied, the effective half-life of the radiopharmaceutical, the dose rate of the exposure, and the repair rate of the studied cells. After continuous internal irradiation from ^131^I, Lassmann et al. [12] exhibited the highest number of irradiation-induced foci in leukocytes 2 h after administration (0.2 excess foci per nucleus). Afterwards, despite subsequently increasing absorbed dose, the foci count decreased. However, Eberlein et al. [13] showed a linear relation between the absorbed dose to the blood and the average number of radiation-induced γ-H2AX foci per cell for peripheral blood lymphocytes up to 5 h after administration of ^177^Lu labeled DOTATATE/DOTATOC, with 0.55 excess foci per cell at 4 h for the average absorbed dose of 34 ± 13 mGy. In all, more systems of cells and variations of irradiations must be studied before a clear dose–effect relationship can be formulated.

When studying a variety of cells within a tissue, cells can be active in the cell cycle, wherein duplication of DNA occurs and radiosensitivity varies. It is well known that ionizing radiation may activate cell cycle checkpoints, which may delay the movement of cells through the cell cycle phases. Cells in the G2/M phase will, per unit absorbed dose, express double the number of γ-H2AX foci than cells in the G_1_/G_0_ phase, consistent with the doubling of DNA content [5]. Our results, obtained at two time points after ^111^InCl_3_ administration, are limited by not knowing the underlying cell cycle progression and possible alteration of progression, which may affect the results seen 25 h P.I.

### Absorbed dose

The calculated average absorbed doses to the testis, assuming a mono-exponential elimination, was 20 mGy for animals 4 h P.I. and 0.1 Gy for animals 25 h P.I. If no sacrifice had been carried out, the total absorbed dose to the testis would have been 0.4 Gy, which is the median lethal dose (LD_50_) for mice spermatogonia [29]. The human testis is much more sensitive, where 0.15 Gy causes a pronounced depression of sperm counts (oligospermia), and temporary sterility (azoospermia) has been reported at 0.3 Gy [25, 26]. However, no significant increase in the number of γ-H2AX foci were seen in the mice exposed to these absorbed doses. However, within the liver of the same mice, the absorbed doses gave rise to a significant increase in the number of γ-H2AX foci per nuclei, even though the absorbed doses to the liver, 0.5 Gy and 3.2 Gy for animals exposed for 4 h and 25 h, respectively, were small in comparison to the absorbed doses held accountable for irradiation effects within the human liver. If no sacrifice had been carried out, the total absorbed dose to the liver would have been 12 Gy, which still is far below the 30–35 Gy from which a 5% incidence of RILD has been observed within the human liver [15, 37, 38].

In this study, because no complete biokinetic data was collected, the absorbed dose calculation accounted only for self-absorbed doses in the testis and the liver itself; it did not include any cross-dose component originating from activity in the surrounding tissue. For both the testis and the liver, the self-absorbed dose is considered a good estimate of the total absorbed dose. It is known that ^111^In is taken up within the tubuli of the testis, more specifically in the basal layer containing the spermatogonia, after systemic administration of ^111^InCl_3_ [16, 17, 33]. Absorbed doses from such a heterogeneous activity distribution can be accounted for using small-scale dosimetry models. For the human testis [41], the self-absorbed dose from ^111^In to the layer of spermatogonia could be a factor of two higher than the corresponding average absorbed dose to the whole testis. In addition, since ^111^In is a radionuclide emitting low energy conversion electrons and Auger electrons with high linear energy transfer (LET), the cellular and subcellular distribution will affect the absorbed dose to the nuclei. Rao et al. have shown a likely intranuclear distribution of ^111^In radiopharmaceuticals within the testicular cells and shown that the subcellular decay sites of high LET Auger-electron emitters primarily determine their radiotoxicity [31, 33]. An intranuclear localization of ^111^In, with decay sites close to the DNA, would further increase the absorbed dose to the DNA. Furthermore, within the liver, published studies [52–54] have shown a heterogeneous radiopharmaceutical distribution after intravenous injections, where radiolabeled colloids such as metal–plasma protein complexes [17, 20–22] tend to accumulate within the liver macrophages, i.e., Kupffer cells. According to a small-scale anatomical model of the human liver tissue for radiation dosimetry [42], the locally absorbed dose close to the source of the activity (e.g., the Kupffer cells) would increase slightly (5–10%) for 10% of the hepatocytes, whereas the self-absorbed dose to the Kupffer cells themselves would be 25 times higher than the average absorbed dose. Hence, a non-uniform radionuclide distribution within the studied tissues is likely present, both on a cellular and on a subcellular level. This, in combination with the high LET from the Auger emissions from ^111^In, may result in a non-uniform absorbed dose distribution to the different cells within the tissues, which may affect the accuracy of our results.

### γ-H2AX immunofluorescence labeling methods and quantitative analyses

In this study, the two different primary antibodies used to target the γ-H2AX epitope showed identical labeling results that are in agreement with the results described in different previous reports [3, 35, 36]. There may be room for certain experimental improvements; for example, the amount of detergent and dilutions used for the antibody incubations can be optimized, thereby influencing antibody targeting. Additionally, to account for the large variation in cellular characteristics in different parts of the testis tubuli and to better understand the differences seen between the testis and the liver, γ-H2AX immunofluorescence labeling could be used for double immunofluorescence labeling, for instance, for correlations with specific cellular phenotypes, apoptosis/cell death, and cell proliferation. This may add both spatial and temporal information about the relation of γ-H2AX to specific cell types and of DSBs in other cellular mechanisms.

Automated image digital analyses for identification of structures in biological tissue samples still require the operator to supply criteria and define regions of interest for the analysis to be carried out, which will, as discussed by Ivashkevich et al. [10], inevitably affect the outcome of the segmentation and hence the counting of nuclei and foci. Therefore, all cell-specific analyses in this work were performed with strong criteria in terms of histology and morphology, and no true automatic segmentation method is presented. The random selection of testis tubuli for the CLSM intranuclear analysis of γ-H2AX foci was performed after localization of high-intensity γ-H2AX-positive tubuli in both exposed and non-exposed animals. This was further motivated by the purpose of the analysis, to investigate whether any divergence between exposed and non-exposed animals was detectable. Hence, only tubular regions with high-intensity γ-H2AX foci regions from the two groups of animals were included in the selection. For further optimization of the quantification method, use of a grid system for random selection of tubuli may be helpful.

For future research, it would be of interest to use high spatial resolution autoradiography techniques to study the corresponding micro-distribution of the radionuclides, which could provide valuable information for more detailed absorbed dose calculations using small-scale dosimetry models. Furthermore, it would be of interest to study the spatial and temporal distribution of γ-H2AX foci throughout larger tissue areas with our suggested method. These could then be correlated to data on a macroscopic level with recently developed PET- or SPECT-imaging of DNA damage repair proteins [55]. Together, these techniques could further refine our understanding of dose–effect relationship.

## Conclusion

The aim of this study was to investigate the possibility of applying the now well-established γ-H2AX method for basic research in the nuclear medicine field. The presented methodology offers a high specific intracellular CLSM method for cell specific γ-H2AX foci quantification. Our methods are potentially applicable to the study of cellular and intracellular radiation-induced effects in different tissues. In this study, the method was exemplified by the testis and liver after systemic administration of ^111^InCl_3_. The methodology might serve as a powerful tool to study the irradiation toxicity of other animal tissues, as well as of human organs (biopsies). With the increasing clinical use of targeted radionuclide therapy, our developed and validated method offers an appropriate tool for studying basic dose–effect relationships in the testicle, liver, and other organs. The results also emphasize the question on the complex link between absorbed dose and biological effects, a primary issue of focus in ongoing radiation protection research.

## Data Availability

The datasets generated during this study are available from the corresponding author on appropriate request.

## Abbreviations

%IA/g: Percent injected activity per gram tissue
2D: Two dimensional
3D: Three dimensional
BSA: Bovine serum albumin
CLSM: Confocal laser scanning microscopy
DAPI: 4′,6-Diamidino-2-phenylindole
DSB: Double-strand break
LET: Linear energy transfer
P.I.: Post injection
PBS: Phosphate buffer saline
PBSTX: Phosphate buffer saline with Triton X
PMT: Photomultiplier tube
RILD: Radiation-induced liver disease
RT: Room temperature
SD: Standard deviation
